# Native Collagen II Relieves Bone Impairment through Improving Inflammation and Oxidative Stress in Ageing db/db Mice

**DOI:** 10.3390/molecules26164942

**Published:** 2021-08-15

**Authors:** Rui Fan, Yuntao Hao, Xinran Liu, Jiawei Kang, Jiani Hu, Ruixue Mao, Rui Liu, Na Zhu, Meihong Xu, Yong Li

**Affiliations:** Department of Nutrition and Food Hygiene, School of Public Health, Peking University, Beijing 100191, China; fanruirf@bjmu.edu.cn (R.F.); haoyuntaolly@163.com (Y.H.); liuhappy07@163.com (X.L.); kangjwdt@163.com (J.K.); hujiani95@163.com (J.H.); rx334@163.com (R.M.); liuruipku@163.com (R.L.); summer920503@163.com (N.Z.); xumeihong@bjmu.edu.cn (M.X.)

**Keywords:** bone impairment, db/db mice, Native Collagen II, inflammation, oxidative stress, T2DM

## Abstract

Ageing-related bone impairment due to exposure to hyperglycemic environment is scarcely researched. The aim was to confirm the improvement effects of Native Collagen II on bone impairment in ageing db/db mice, and the ageing model was established by normal feeding for 48-week-old. Then, the ageing db/db mice were randomly assigned to Native Collagen II intervention, the ageing model, and the chondroitin sulfate + glucosamine hydrochloride control groups. After 12 weeks of treatment, femoral microarchitecture and biomechanical parameters were observed, biomarkers including bone metabolism, inflammatory cytokines, and oxidative stress were measured, and the gastrocnemius function and expressions of interleukin (IL) 1β, receptor activator of nuclear factor (NF)-κB ligand (RANKL), and tartrate-resistant acid phosphatase (TRAP) were analyzed. The results showed that the mice in the Native Collagen II intervention group showed significantly superior bone and gastrocnemius properties than those in the ageing model group, including bone mineral density (287.65 ± 72.77 vs. 186.97 ± 32.2 mg/cm^3^), gastrocnemius index (0.46 ± 0.07 vs. 0.18 ± 0.01%), muscle fiber diameter (0.0415 ± 0.005 vs. 0.0330 ± 0.002 mm), and cross-sectional area (0.0011 ± 0.00007 vs. 0.00038 ± 0.00004 mm^2^). The Native Collagen II intervention elevated bone mineralization and formation and decreased bone resorption, inflammatory cytokines, and the oxidative stress. In addition, lower protein expression of IL-1β, RANKL, and TRAP in the Native Collagen II intervention group was observed. These findings suggested that Native Collagen II improved bones impaired by T2DM during ageing, and the likely mechanism was partly due to inhibition of inflammation and oxidative stress.

## 1. Introduction

Diabetes mellitus (DM), caused by dysfunctional glucose metabolism, is a chronic endocrine disorder. Type 2 DM (T2DM) is a multifactorial disease, the central pathomechanisms of which are hyperinsulinemia, insulin resistance, and chronic inflammation, and progressive secondary β-cell failure occurs at the later stages [1,2]. With the increasing duration of DM, patients suffer from the complications including retinopathy, nephropathy, neuropathy, and vascular disease. DM accelerates material and microstructural bone deficits, finally leading to some bone diseases, including osteoporosis and osteoarthritis [3,4,5]. Now, bone damage is known as a complication of DM [6], which is associated with significant morbidity, mortality, and reduction in quality of life [7]. Hence, the development of effective management of promoting early intervention and prevention of bone degeneration to improve the quality of life among elderly T2DM patients is needed.

There is a close relevance between bone and glucose metabolism [8]. Osteocalcin is a good example; it is produced by osteoblasts and odontoblasts and also plays an important role as an endogenous insulin sensitizer [9]. Thus, bones are both influenced by glucose metabolism and are capable of modulating it [10]. T2DM was reported to impair bone health by unbalancing a series of processes including bone formation and resorption, collagen formation and crosslinking due to hyperglycemia, high level of reactive oxygen species (ROS), inflammation and advanced glycation end products (AGEs) [11,12,13,14]. In addition, insulinopenia and lack of IGF-1 also influenced osteoblasts [15,16]. It is well-known that skeletal muscles are the main site for insulin-mediated glucose disposal and energy metabolism. It has been reported that persons with DM have accelerated muscle loss [4]. In fact, skeletal muscles and bones have a coupled and cross-talk relationship, which shows the secreting myokines including IGF-1 and acting for monokine communication [17]. The accumulation of these pathomechanisms ultimately leads to decreased bone quality in T2DM. Therefore, what we need to improve is the understanding of the factors that determine bone health in people with T2DM, especially in elderly patients. The current research revealed the mechanism of influence of Native Collagen II on bone loss in ageing db/db mice in the musculoskeletal system for the first time.

Collagen, the main component of bones and cartilages, possesses an important function. Type II collagen, the main part of cartilages, is an interconnected network of collagen and proteoglycans that are crucial in maintaining joint flexibility and resistance to stress and fractures [18,19]. Several studies have suggested that a diet with a high percentage of collagen peptides improves bone collagen metabolism and the complications of hyperglycemia [20,21]. According to many reports, type II collagen can treat arthritis due to its anti-inflammatory properties [22,23]. Native Collagen II, showing intact biological activity, receiving much attention [24], was reported to be effective in rheumatoid arthritis and arthritis [25,26] due to reducing joint inflammation and promoting cartilage repair [27,28]. The potential mechanism is due to the oral tolerance. When consumed, Native Collagen II is believed to be taken up by Peyer’s patches, where it activates immune cells. When they recognize type II collagen in joint cartilages, Treg cells secrete anti-inflammatory mediators (cytokines). This action helps reduce joint inflammation [29]. Unexpectedly, a prior report found Native Collagen II could augment bone mineral density, bone volume, and trabecular number, decrease trabecular separation, and improve intense bone resorption, which aids in the maintenance of cancellous bone, although this was a non-significant trend [30].

On the basis of the role of various cytokines in T2DM coupled with the previous findings, it is speculated that supplementation with Native Collagen II could relieve bone impairment among T2DM patients. In order to simulate a real situation of bone impairment during the developing process of T2DM, 48-week-old db/db mice normally fed with a basic feed were used as the ageing model, a model established for the first time in this study. We investigated the effects of Native Collagen II on bone quality, microstructure, biomechanics, and metabolism properties in ageing db/db mice and explored the underlying mechanisms with a comprehensive and systematic perspective of the musculoskeletal system for the first time. This study could identify possible evidence of nutritional solutions for bone impairment in the future research on elderly T2DM patients.

## 2. Results

### 2.1. General Condition and Disease State

The mice in the NC group exhibited smooth, shiny, and lustrous hair, energy, and fecal columnar formation, whereas the mice in the AM and YM groups featured weak, coarse, and dull hair, unresponsiveness, and decreased activity. Compared with the AM and YM groups, the CG and UC groups showed more activity.

**Figure 1:**
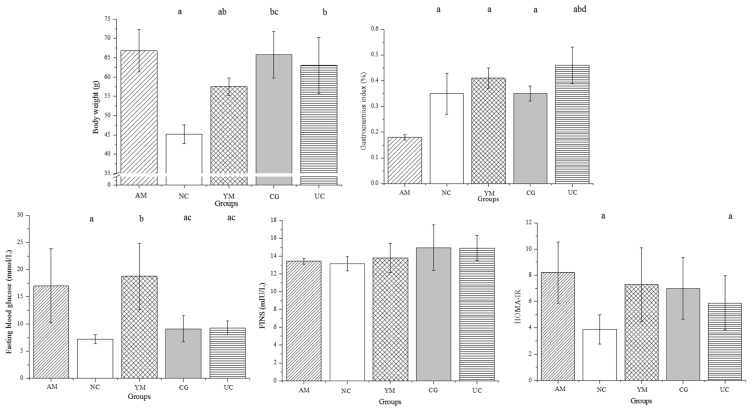
The effect of Native Collagen II on the body weight, gastrocnemius index, plasma glucose, and insulin level in the five groups. The data are the means ± SD, (*n* = 5); ^a^ *p* < 0.05 versus the AM group, ^b^ *p* < 0.05 versus the NC group, ^c^ *p* < 0.05 versus the YM group, ^d^ *p* < 0.05 versus the CG group.

Figure 1 shows the body weight; the weight in the AM group was significantly larger than in the YM and NC groups (*p* < 0.05), while the body weight difference between the AM and intervention (CG and UC) groups showed no statistical significance (*p* > 0.05). The gastrocnemius index in the AM group was the lowest, which presented significant differences between the NC, YM, CG, and UC groups (*p* < 0.05). The gastrocnemius index in the UC group was significantly higher than in the NC and CG groups (*p* < 0.05), which indicated that Native Collagen II resulted in an obvious increase in the gastrocnemius index.

FBG levels were less than 9.0 mmol/L in the CG and UC groups, i.e., lower than those in the AM and YM groups (>15 mmol/L; *p* < 0.05), but the levels were slightly larger than in the NC group (<7.5 mmol/L; *p* > 0.05). There was no significant difference in FINS between the different groups. Homeostatic model assessment of insulin resistance (HOMA-IR) is an index that evaluates the level of insulin resistance. The NC group showed the lowest HOMA-IR, and the HOMA-IR index of the UC group was significantly lower than in the AM group (*p* < 0.05).

### 2.2. Micro-CT Femoral Analysis

**Figure 2:**
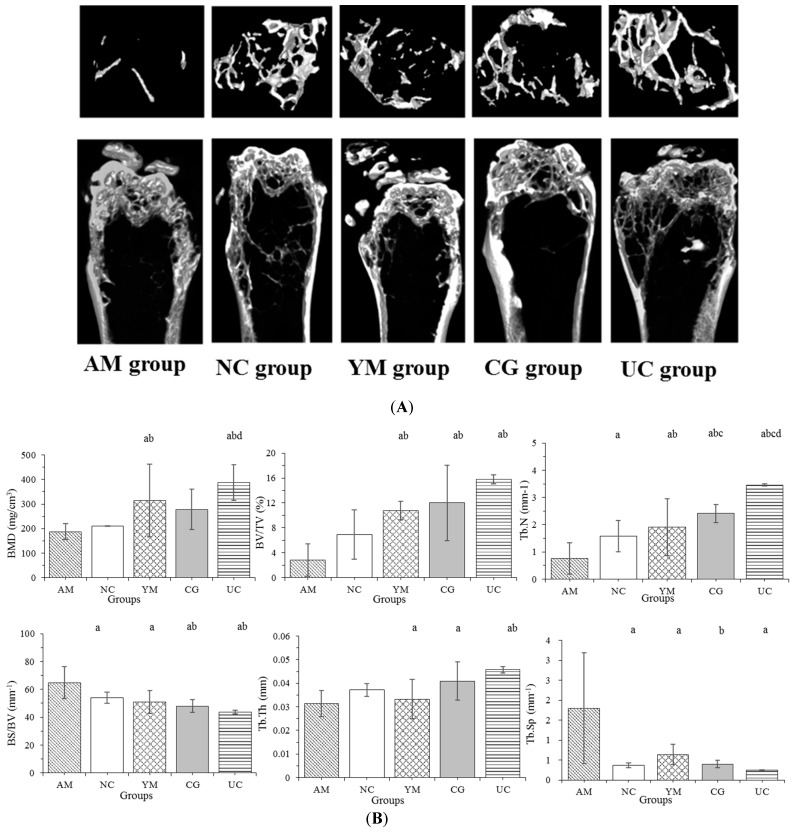
The histomorphometry of the femur in the five groups. (**A**) The images of the changes in bone microarchitecture. (**B**) The mineral density and histomorphometry of the femur. The data are expressed as the means ± SD (*n* = 5 in the AM group, *n* = 7 in the YM, CG, NC, and UC groups); ^a^ *p* < 0.05 versus the AM group, ^b^ *p* < 0.05 versus the NC group, ^c^ *p* < 0.05 versus the YM group, ^d^ *p* < 0.05 versus the CG group.

Micro-CT imaging was performed to quantitatively measure the bone mass on the basis of BMD and the most common microarchitectural parameters of cancellous bone including: BV/TV, Tb.N, Tb.Th, Tb.Sp, which reflect bone microvolume, trabecular bone number, thickness, and spacing, respectively. Micro-CT analysis results in Figure 2 show that the effects of Native Collagen II on femoral BMD and histomorphometry were significant. BMD values in the AM and NC groups were relatively small and significantly smaller than those in the YM and UC groups (*p* < 0.05). BMD in the UC group was the largest among the five groups, 2.07, 1.84, and 1.39 times higher compared with those values in the AM, NC, and CG groups, respectively. BV/TV represents the fraction of a given volume of interest, total volume, or tissue volume that is occupied by bone. The effect of UC on the BV/TV level was similar to that on BMD, which exhibited a significantly larger level in the YM, CG, and UC groups than in the AM and NC groups (*p* < 0.05). For BS/BV, the level order was as follows: AM group > NC group > YM group > CG group > UC group, with the BS/BV values in the AM group significantly larger than in other groups (*p* < 0.05); the values in the CG and UC groups were significantly smaller than those in the AM and NC groups (*p* < 0.05). In addition, the smallest values of Tb.Th and Tb.N were observed in the AM group, and they significantly differed from those in the CG, YM, and UC groups (*p* < 0.05). The largest value of Tb.N was observed in the UC group, which was significantly different from that in the other groups (*p* < 0.05), while the AM group showed the largest values of Tb.Sp, which were significantly larger than those in other groups (*p* < 0.05).

### 2.3. Dynamic Histomorphometric and Biochemical Markers of Bone Turnover Analysis

**Figure 3:**
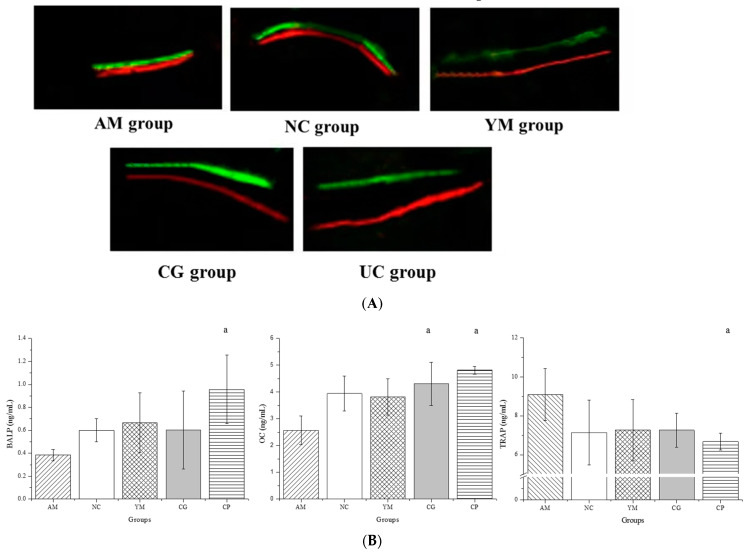
Dynamic histomorphometric and biochemical markers of bone turnover analyses. (**A**) Dynamic histomorphometric analyses of representative fluorescence images (*n* = 5); (**B**) serum levels of biochemical markers of bone turnover (*n* = 5, 7, 9, 9, and 10 in the AM, CG, UC, NC, and YM groups); ^a^ *p* < 0.05 versus the AM group.

Dynamic histomorphometric analyses of representative fluorescence images obtained from the femur are shown in Figure 3A. The green fluorescence in the images is calcein green, which represents the first mineralization label; the red fluorescence in the images is alizarin complexone, which represents the second mineralization label. The width of the gap between the red and the green fluorescence in the images could, to some extent, represent the bone formation rate. Relatively larger gap widths were observed in the UC group, and relatively smaller ones were, remarkably, observed in the AM and NC groups, which indicated that Native Collagen II treatment led to bone formation, which was consistent with the results of bone microarchitecture (Figure 3).

Bone formation and bone resorption determined bone turnover [31]. In addition, the serological assessment of bone turnover in the five groups is shown in Figure 3B. AGEs play an important role in the bone diseases development (such as osteoporosis), especially among T2DM patients. Their accumulation in the bone alters osteoblasts, inducing enhanced osteoclastogenesis and impaired matrix mineralization (downregulation of alkaline phosphatase and osteocalcin mRNA) [32]. OC, mainly controlling mineralization, and BALP, representing bone formation, were selected. TRAP, a marker of osteoclast numbers, was selected to represent bone resorption on the basis of a previous report [30]. As expected, the serum level of BALP in the UC group was obviously larger than in the AM, NC, YM, and CG groups (*p* < 0.05), which was consistent with the histomorphometric bone analysis. Similarly to the BALP data, the OC serum content in the OM group was significantly lower than in the UC and CG groups (*p* < 0.05). Moreover, TRAP serum levels were the lowest in the UC group (*p* < 0.05).

### 2.4. Bone Biomechanical Parameter Analysis

The three-point bending technique was used to assess the mechanical properties (fracture point, stiffness, and elasticity) of the femur. A constant amount of force was applied to the midpoint of the femur diaphysis to determine the maximum load that could be placed on the femur until fracture/breakage occurred. Table 1 shows biomechanical femoral parameters in the five groups. The values of the maximum load in the CG and UC groups were significantly larger than those in the AM and NC groups (*p* < 0.05), of which the value in the UC group was significantly larger than that in the YM group (*p* < 0.05), and biomechanical femoral properties, including the energy to ultimate load, Young’s modulus, stiffness, and breaking energy in the UC group showed significant differences between the AM, NC, YM, and CG groups (*p* < 0.05).

### 2.5. Inflammation and Oxidative Stress in the Serum

**Figure 4:**
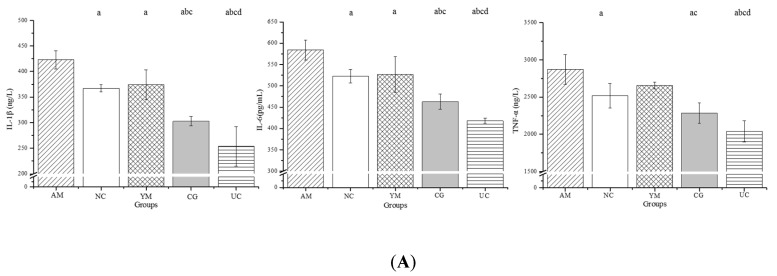

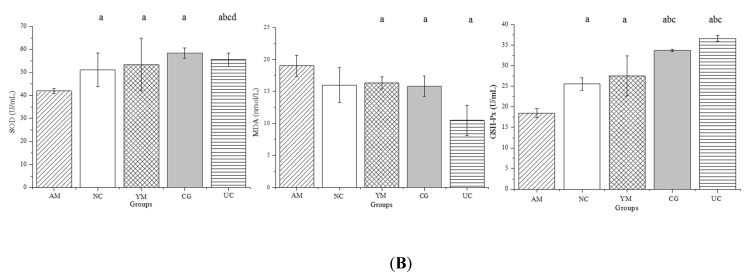
Serum levels of inflammatory cytokines and oxidative stress in the five groups. (**A**) Serum levels of inflammatory cytokines including IL-1β, IL-6, and TNF-α; (**B**) serum levels of oxidative stress indexes including MDA, SOD, and GSH-Px. Serum data are expressed as the means ± SD (*n* = 5, 7, 9, 9, and 10 in the AM, CG, UC, NC, and YM groups); ^a^ *p* < 0.05 versus the AM group, ^b^ *p* < 0.05 versus the NC group, ^c^ *p* < 0.05 versus the YM group, ^d^ *p* < 0.05 versus the CG group.

It was approved that increased production of adipocytes and hyperglycemia feed the cycle of chronic inflammation by producing ROS and inflammatory cytokines, which induce osteoblast apoptosis to bring the negative effect on bone health. In the current study, to assess the inflammation and oxidative stress, inflammatory cytokines and the oxidative stress reaction were investigated. The serological assessment of inflammatory cytokines in the five groups is shown in Figure 4A. Similar change trends were observed for IL-1β, IL-6, and tumor necrosis factor (TNF) α. The levers of IL-1β, IL-6, and TNF-α in the NC, YM, CG and UC groups were significantly smaller in the OM group (*p* < 0.05). In addition, IL-1β, IL-6, and TNF-α levels in the UC group were significantly smaller than those in the other groups (*p* < 0.05).

In order to investigate the effect of Native Collagen II on oxidative stress, the activity of superoxide dismutase (SOD), glutathione peroxidase (GSH-Px), and malondialdehyde (MDA) in the serum of the mice was examined. As shown in Figure 4B, compared with the AM group, the other groups showed obviously stronger activities of SOD and GSH-Px and a significantly lower MDA level (*p* < 0.05). Regarding the MDA content, values in the UC group were the smallest among the five groups (significantly; *p* < 0.05), whereas there were no significant differences between the SOD and GSH-Px activity found in the two intervention groups (*p* > 0.05).

### 2.6. Histopathological Muscle Analysis and Its Biomarker

**Figure 5:**
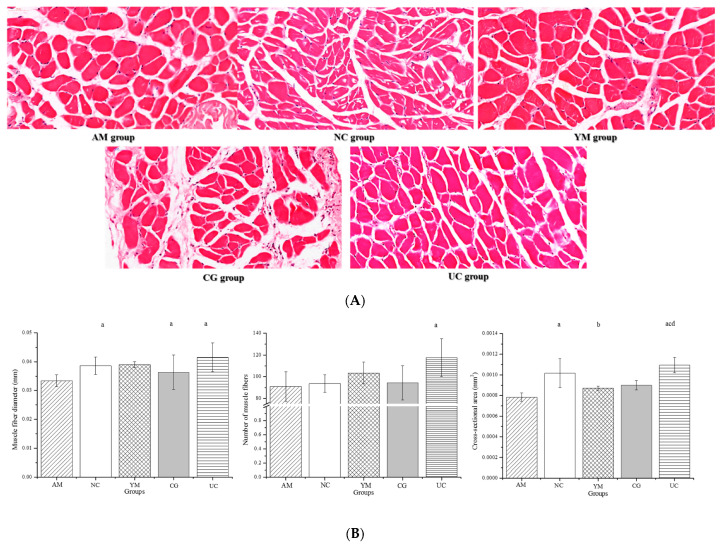

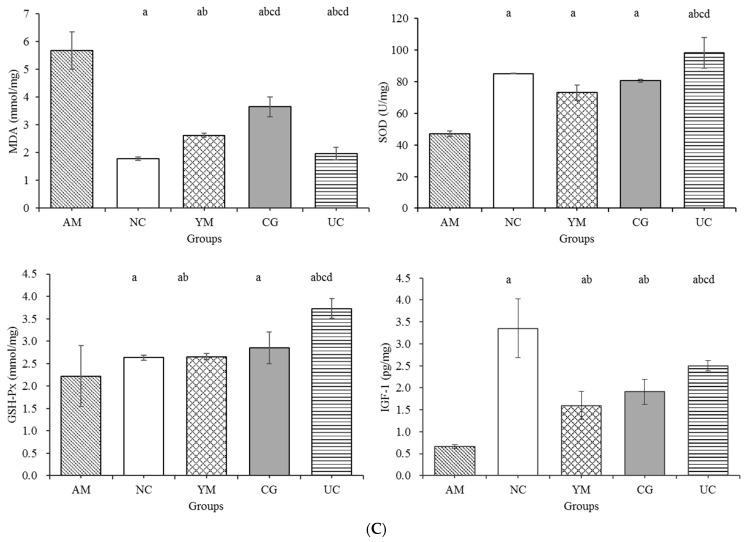
H&E staining of the gastrocnemius muscle and its characteristic parameter. (**A**) H&E staining of the gastrocnemius. The bar indicates 50 μm. Magnification: 40×. (**B**) Diameter, number, and cross-sectional area of muscle fibers. (**C**) Biomarkers including oxidative stress and monokines IGF-1. The data are expressed as the means ± SD, *n* = 5; ^a^ *p* < 0.05 versus the AM group, ^b^ *p* < 0.05 versus the NC group, ^c^ *p* < 0.05 versus the YM group, ^d^ *p* < 0.05 versus the CG group.

We had known that the gastrocnemius decreased in the AM group and the Native Collagen II intervention could increase the gastrocnemius mass. The gastrocnemius morphological characteristics and pathology are shown in the Figure 5A,B. Muscle cells in the AM group lost their normal morphology, the nuclear center migration phenomenon was serious, and the cells showed remarkable differences in size and had a disordered, loose arrangement. In addition, the intercellular substance level increased, and there was a large amount of connective-tissue hyperplasia and inflammatory cell infiltration between muscle cells. Similarly to the AM group, the intercellular substance level in the CG group increased, and there was a large amount of fibrous connective-tissue hyperplasia and inflammatory cell infiltration; however, it was inconsistent because the CG group showed normal cell morphology, uniform sizes, and a slight nuclear center migration phenomenon. Compared with the AM and CG groups, the NC and YM groups showed less fibrous connective-tissue hyperplasia and inflammatory cell infiltration, and the cells were uniform in size and tidily arranged. UC resulted in a normal polygonal cell without degenerating necrotic cells, uniform size, tightly arranged, and with the disappearance of disorderly connective tissue and inflammatory cells.

Impaired glucose/insulin metabolism may indirectly affect bones and muscles by altering skeletal muscle signaling, such as IGF-1. It was reported that IGF-1 was associated with increasing the muscle mass and the muscle fiber area. Figure 5B shows the effect of Native Collagen II on muscle fiber parameters in the five groups. The AM group showed the smallest values in muscle fiber diameter, number, and cross-sectional area, while the intervention with Native Collagen II significantly increased the muscle fiber diameter, number, and cross-sectional area (*p* < 0.05). The improvement effect of positive substances (CS + GH) on the muscle fiber cross-sectional area was smaller compared with that of the Native Collagen II intervention.

IGF-1 is known to activate IGF-1R which acts through the PI3K/Akt and the MAPK/ERK pathways [33]. It has been speculated the function of IGF-1 was partly affected by inflammation, oxidative stress, and diabetes [34]. In order to investigate the effect of Native Collagen II on oxidative stress in the gastrocnemius muscle of the db/db mice, the activity of SOD, GSH-Px, and MDA in the gastrocnemius muscle was determined. According to Figure 5C, the activities of SOD and GSH-Px in the AM group were significantly lower than those in four other groups, and the UC group showed a significantly higher level than that in the other groups (*p* < 0.05), while the MDA content in the UC group was remarkably decreased (*p* < 0.01), indicating that the UC intervention exerted protection against SOD and GSH-Px depletion and MDA accumulation in the gastrocnemius muscle tissue of the db/db mice.

IGF-1, secreted from skeletal muscles, was an important growth factor for skeletal development [35]. For the IGF-1 levels, the lowest content was found in the AM group, which was obviously lower than that in the other groups (*p* < 0.05); an intervention with Native Collagen II increased the IGF-1 content, which was higher than in the AM, YM, and CG groups and lower than in the NC group (*p* < 0.05).

### 2.7. Immunohistochemical Analysis

**Figure 6:**
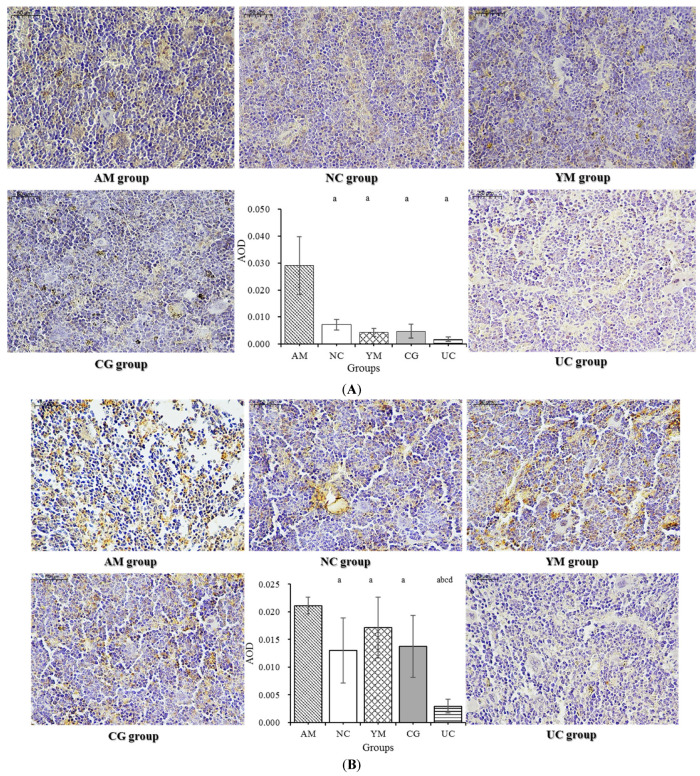

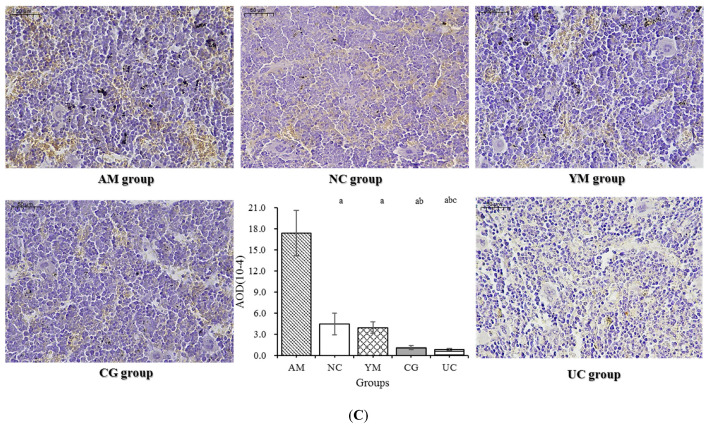
Immunohistochemistry analysis of the five groups. Magnification: ×400. IHC quantification is shown in the bar graphs. (**A**) Protein expressions of IL-1β in the femur; (**B**) protein expressions of RANKL in the femur; (**C**) protein expressions of TRAP in the femur. The data are the means ± SD (*n* = 5, 7, 9, 9, and 10 in the AM, CG, UC, NC, and YM groups); ^a^ *p* < 0.05 versus the AM group, ^b^ *p* < 0.05 versus the NC group, ^c^ *p* < 0.05 versus the YM group, ^d^ *p* < 0.05 versus the CG group.

It was reported that some proinflammatory cytokines, including IL-1β, IL-6, and TNF-α, also stimulate osteoclast activity by the actions of RANKL [36]. RANKL, an essential factor for osteoclast differentiation and function, mediates bone loss by promoting the bone-resorbing activity of osteoclasts and prolongs their survival [37,38]. The receptor activator of (nuclear factor) kappa B ligand (RANKL) signaling pathway was analyzed. Figure 6A shows that the immunostaining of IL-1β in the AM, NC, YM, and CG groups was stronger than in the UC group. The areal density of IL-1β in the AM group was significantly higher than in the four other groups (*p* < 0.05), which indicated that IL-1β in the AM group was remarkably more strongly expressed, while the Native Collagen II intervention significantly decreased the expression of IL-1β (*p* < 0.05). The RANKL expression is shown in Figure 6B. A higher expression in the AM group was clearly detected, and there was little expression in the NC and UC groups. Compared with the areal density in the AM group, it was significantly decreased in the four other groups (*p* < 0.05). AOD in the UC group was obviously lower than in the AM, NC, YM, and CG groups (*p* < 0.05). Figure 6C shows that the immunoreactive signal for TRAP in the UC group was weaker than that in the other groups. AOD indicated that the highest expression was in the AM group, while the lowest expression was in the UC group; TRAP expression in the UC group was relatively lower than in the AM, NC, and YM groups (*p* < 0.05).

## 3. Materials and Methods

### 3.1. Materials

Native Collagen II, with the conformational integrity of the triple-helical structure remaining intact, was purchased from SEMNL Biotechnology Co. Ltd. (Beijing, China). Native Collagen II, prepared from a chicken sternum at a low temperature, showed the molecular weight of 300 kDa, and its content was as follows: collagen, 263.0 mg/g; hydroxyproline, 32.9 mg/g. The triple-helical structure and the second structure (17.2% of the α helix, 21.1% of the β sheet, 44.0% of the β turn, and 17.1% of the nonregular coil) are shown in the Supplementary Materials. The amino acid composition is shown in Table 2. Chondroitin sulfate (CS) and glucosamine hydrochloride (GH) were obtained from Wilke Resources (Lenexa, KS, USA).

The detection kits of superoxide dismutase (SOD), glutathione peroxidase (GSH-Px), malondialdehyde (MDA), tumor necrosis factor α (TNF-α), interleukin (IL) 6 and IL-1β were purchased from Shanghai Lengton Biotechnology Co., Ltd. (Shanghai, China). Insulin was bought from the Beyotime Institute of Biotechnology (Beijing, China). The detection kits of alkaline phosphatase (BALP), tartrate-resistant acid phosphatase (TRAP), and osteocalcin (OC) were bought from USCN Life Science Inc. IL-1β, IL-6, TRAP, and receptor activator of nuclear factor (NF)-κB ligand (RANKL) antigens (mouse monoclonal antibody) were purchased from R&D Systems, USA. The basic feed, which met the national standard (GB 14924.3-2010), was bought from Beijing Keao Xieli Co. Ltd. (Beijing, China). All the other reagents were analytically pure.

### 3.2. Animals and Experiment Design

#### 3.2.1. Animal Feeding Conditions and Ageing db/db Mice Model Establishment

Male C57BL/KsJ-leprdb/leprdb diabetic (db/db, 10-week-old) mice and male nondiabetic littermate (db/m) mice were obtained from the Animal Service of the Health Science Center (Peking University, Beijing, China), production certificate No. SCXK (Beijing, China) 2016–0010, use license No. SYXK (Beijing, China) 2016-0041. The environment conditions involved constant temperature (21–25 °C), relative air humidity (50–60%), and 12 h light/dark cycles.

All the mice had free access to the standard food (American Institute of Nutrition Rodent Diet 93G) and water. The protocols were reviewed and approved by the Institutional Animal Care and Use Committee of Peking University (approval No. LA2015094).

Considering bone impairment associated with T2DM and ageing, we used, for the first time, 48-week-old db/db mice normally fed with a basic feed representing the approach of ageing as the ageing model to explore the effect of the Native Collagen II intervention begun in their later life on the ageing-related bone impairment, meanwhile, 25-week-old db/db (the age considered to represent the end of growth and development) mice normally fed with a basic feed as the young model to explore the alterations of bone impairment during the process of T2DM and ageing, which could lead to a deeper understanding of the occurrence and development of bone impairment in T2DM. Consequently, the ageing and the young models of db/db mice is the highlight of this work (the timepoints were selected on the basis of previous research coupled with the observation of the general condition and the disease state including intake, drinking, weights and fasting blood glucose provided in Figures S3–S5 from the Supplementary Materials) [39,40].

#### 3.2.2. Study Design

Forty-five 48-week-old db/db mice (72 ± 3 g) with plasma glucose above 11.1 mmol/L were randomly subdivided into the ageing model group (AM; *n* = 15), the Native Collagen II intervention group (UC; *n* = 15), and the chondroitin sulfate + glucosamine hydrochloride (CS + GH) control group (CG; *n* = 15). Meanwhile, 48-week-old db/m and 25-week-old db/db mice were used as the normal control (NC; *n* = 15) and the young model (YM; *n* = 15) groups, respectively. The UC group was administered Native Collagen II through drinking at the dose of 6 mg/kg body weight, whereas the CG group was treated with 180 mg CS + 225 mg GH/kg body weight through drinking. The AM, NC, and YM groups were administered distilled water. The drinking feeding mode was a gentle feeding mode for elderly mice, whose strict quality control and accuracy were confirmed by our team with solid results in earlier reports [41,42].

After dividing the group, the general condition, including the coat color, mental state, and daily activities, was monitored daily (at 10:00) till the end stage of the experiment, and food intake, water intake, and body weight were regularly recorded each week.

**Figure 7:**
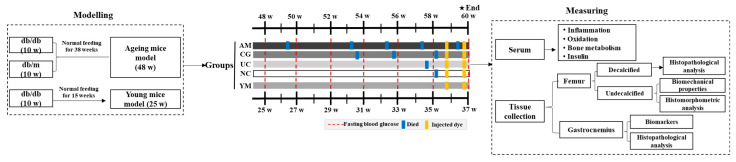
Schematic plot and the timeline of the research protocol.

All the mice were exposed to the intervention for 12 weeks. In the later stage of the intervention period, weight loss showing a sharp decrease (approximately >20% loss of body weight) was an indication of the dying stage, and if a mouse appeared to be dying due to weight loss or injury, it was euthanized with CO_2_ to minimize suffering. One-quarter of all the mice or one-third of one group approaching death were considered to signify the end of the experiment (here, the intervention lasted 12 weeks). At the end of the experiment, the surviving mice (*n* = 10, 13, 14, 14, and 15 in the AM, CG, UC, NC, and YM groups, respectively) were euthanized with CO_2_, and blood was obtained from the retrobulbar plexus using heparinized anticoagulant tubes for further measurements. After euthanasia, the main organs were weighed to calculate organ coefficients. Some separated right femurs from the mice injected with a dye (*n* = 5 in each group) were fixed in 10% neutral buffered formalin for dynamic histomorphometry; the left femurs of these mice were packed in a gauze soaked with phosphate-buffered saline and stored at –20 °C to carry out the three-point test; the right gastrocnemius of these mice was separated and stored at –80 °C to perform biomarker measurements, including oxidative stress and myokines; and the left gastrocnemius of these mice was fixed in 10% neutral buffered formalin for histopathological analysis. The mice without an injection in each group provided serum and femurs to measure biomarkers, and for microcomputed tomography (CT) and histopathological analysis; histopathological analysis for the right femur also required decalcification with 10% EDTA (changed each week). The schematic diagram of the research protocol is shown in Figure 7.

Normal deaths were marked with blue. Injection of alizarin-3-methyliminodiacetic acid and calcein was performed in one-week intervals (*n* = 5 mice per group per timepoint) (marked with yellow). Fasting blood glucose and insulin measurements were performed each week (in all the survived mice per group per timepoint) and at the end stage (*n* = 5 injected mice per group).

### 3.3. Fasting Plasma Glucose and HOMA-IR Measurements

Fasting plasma glucose (FBG) and insulin (FINS) in the tail vein were measured using an Accu-Check glucometer (Roche Diagnostics) and an ELISA kit after fasting for 5 h (*n* = 5 injected mice of each group). The level of insulin resistance was calculated using the following formula: (fasting glucose (mmol/L) × FINS (uIU/mL))/22.5 [43] and expressed as HOMA-IR.

### 3.4. Micro-CT Analysis

The mass and microarchitecture of undecalcified femurs were measured with micro-CT (Inveon MM system, Siemens, Munich, Germany), and the parameters were calculated using an Inveon Research Workplace. The parameters, including 8.82 μm for pixel size, 80 kV for voltage, 500 μA for current, and 1500 ms for exposure time were set. The trabecular region of about 1–2 mm distal to the proximal epiphysis was selected. Under these conditions, the parameters including bone volume/total volume (BV/TV), bone surface area/bone volume (BS/BV), trabecular thickness (Tb.Th), trabecular number (Tb.N), trabecular separation (Tb.Sp), and bone mineral density (BMD) were obtained.

### 3.5. Biomechanical Bone Parameters

In order to evaluate the biomechanical properties of the femurs, three-point bending tests were performed. The left femurs of the injected mice in each group were centrally loaded with a speed of 1 mm/s using a universal testing machine (Instron 4501, Instron, Canton, MA, USA). The parameters of the bone samples, namely, stiffness, ultimate strength, Young’s modulus, and ultimate stress were determined and calculated based on the deformation curve [44].

### 3.6. Slicing of Undecalcified Bones and Dynamic Histomorphometric Analysis

Seven days before euthanasia, five mice per group were intraperitoneally injected with alizarin red (30 mg/kg body weight); 4 days later, an intraperitoneal injection of 20 mg/kg body weight calcein was carried out [45]. After euthanasia, the right femurs were fixed and dehydrated and embedded in destabilized methyl methacrylate resin. The samples were ground and polished to 40–60 μm and H&E stained. The samples were then observed under a laser scanning confocal microscope (Leica TCS SP-2, Frankfurt, Germany) set on 534 nm (calcein) and 357 nm (alizarin red) [46].

### 3.7. Biochemical Marker Assay

TRAP, a marker of the osteoclast number, represented bone resorption. OC, mainly controlling for mineralization, and BALP, representing bone formation, were selected. The levels of BALP, TRAP, OC, common inflammatory cytokines (TNF-α, IL-1b, IL-6), and common oxidative stress markers (SOD, GSH-Px, MDA) in the serum of the mice without a dye injection were measured using relevant enzyme-linked immunoassay (ELISA) kits according to the product instructions.

SOD, MDA, GSH-Px, and IGF-1 activities in the right gastrocnemius muscle (*n* = 5 in each group) were measured using the relevant ELISA kits according to the product instructions.

### 3.8. Histopathological Analysis

The left gastrocnemius of the five injected mice in each group was fixed in 10% formalin. The samples embedded in paraffin were stained with hematoxylin and eosin (H&E). In order to evaluate the status of the muscles, gastrocnemius slides were microscopically observed with an inverted microscope (Olympus IX70, Olympus, Tokyo, Japan). Then, the cross-sectional area of muscle fiber diameters was measured and calculated with Image-Pro Plus 6.0 (Media Cybernetics, Inc., Rockville, MD, USA).

Immunohistochemistry (IHC) was used to deduce the involvement of the related key pathways, performed on the right femur of the mice without a dye injection. IL-1β, IL-6, tartrate-resistant acid phosphatase (TRAP), and receptor activator of (nuclear factor) NF-κB ligand (RANKL) were applied with the following antibodies: IL-1β and IL-6 antigens (both 1:100 dilution) for proinflammatory cytokine expression; TRAP (1:100 dilution) and RAKNL (1:100 dilution) antigens for osteoclast formation. Immunohistochemistry was carried out on 3–5 μm-thick paraffin sections, followed by overnight incubation with the primary antibody at 4 °C. After staining with DAB, the slides were counterstained with Mayer’s hematoxylin and dehydrated through a graded ethanol series.

Quantitative image analysis was performed with the Image-Pro Plus 6.0 software (Media Cybernetics, Inc., Rockville, MD, USA). Cumulative optical density (IOD) and pixel area (area) values of the tissue of each positive image were obtained. Statistical analysis of the results was expressed as the average optical density (AOD) (areal density, AOD = IOD/area).

### 3.9. Statistical Analysis

The data are expressed as the means ± SD. One-way ANOVA with post-hoc LSD (equal variances assumed) or Dunnett’s T3 test (equal variances not assumed) were performed with SPSS 18.0, and *p*-values less than 0.05 were considered to be significant.

## 4. Discussion

Diabetes-associated bone disease leads to impaired bone quality and increased fracture risk. This study addressed the alterations of bone quality-impaired T2DM and ageing, including bone microarchitecture, remodelling, and biomechanical quality. In order to evaluate the effect of Native Collagen II on bone quality impairment in the ageing db/db mice and its mechanism of action, we comprehensively explored bone metabolism alterations on the basis of inflammation and oxidative stress in the serum, bone, and skeletal muscle for the first time. In addition, the T2DM status was examined.

BMD values in the AM group were similar to those in the NC group, which was consistent with the previous research finding normal or even mildly elevated BMD in T2DM patients compared to that in those without T2DM [47]. In fact, fracture risk is increased; although there is normal and even elevated BMD in T2DM, the evidence led to the hypothesis that there are diabetes-associated alterations in skeletal properties [48]. In this study, alterations including bone microarchitecture, metabolism, and biomechanical quality caused by T2DM were investigated. The lowest levels of BV/TV, Tb.N, and Tb.Th were in the ageing db/db mice (AM group), which was consistent with the previous results obtained in diabetic rats and humans with T2DM [48,49]; this deteriorated morphological structure was sharply improved by Native Collagen II. Moreover, after the Native Collagen II intervention, the thickness of the cortical bone was observed, and cortical porosity and trabecular heterogeneity were improved. There were morphological and histological changes of bone tissue in this study, which led to significant worsening of the mechanical properties shown in the three-point bending test (Table 1). The mice in the AM group showed the smallest values in the maximum load, the energy to ultimate load, Young’s modulus, stiffness, and breaking energy, which were similar to those in another report [50,51].

In addition, the alterations of the bone metabolism were analyzed. Serum levels of BALP and OC in the AM group were the lowest, whereas its TRAP levels were significantly higher compared to those in the other groups. Similar conclusions were reported in previous studies, both in rodents and people with T2DM [52,53,54], while the increased value in TRAP observed in the AM group was not consistent with other reports [54]. In fact, the studies examining the effect of diabetes mellitus on bone resorption were not conclusive, including unaltered, inhibited, and increased in vitro and vivo studies on animals and human patients [55,56,57,58].

The abovementioned alterations in microarchitecture, mechanical properties, and bone metabolism were easy to understand on the basis of both indirect and direct T2DM effects on bone impairment. A disorder in glucose and insulin metabolism decreases the activity of osteoblasts and osteoclasts. Hyperglycemia changes gene expression associated with osteoblast activity, and insulin resistance may impact bone health [59]. Inflammation and oxidative stress induced by T2DM have negative effects on bone metabolism. These accumulation pathomechanisms ultimately result in decreased bone formation or bone resorption, leading to decreased bone quality [8,35]. In this study, the Native Collagen II intervention relieved bone impairment in ageing db/db mice, reflected in improving the bone microarchitecture, increasing bone formation, and decreasing bone resorption. Native Collagen II also improved inflammation, oxidative stress, and muscle properties, which might have elevated the disease status of T2DM.

What is the possible reason for Native Collagen II improving bone impairment induced by T2DM? A possible mechanism was the decrease in inflammation and oxidative stress, which could directly regulate bone metabolism through bones, and indirectly through muscles and blood glucose.

From a comprehensive and systematic point of view, skeletal muscles and bones have a coupled and cross-talk relationship, where bones act as a lever and muscles act as a pulley to move the organism and for monokine communication. The mice in the AM group exhibited lower values of BV/TV, Tb.N, and Tb.Th and showed smaller values in the muscle mass, muscle fiber diameter, and cross-sectional area (Figure 3 and Figure 6). In addition, impaired glucose or insulin metabolism may impact bones by changing skeletal muscle signaling. IGF-1, secreted from skeletal muscles, is an important growth factor for bone development. Some investigations provided evidence that muscle IGF-1 can modulate bone formation and maintain bone structure, which was correspondingly shown in this study; we found higher content of IGF-1 in the CP group, which showed a higher level of BV/TV and trabecular number (Tb.N) [28,60,61,62,63]. IGF-1 is also an important growth factor for muscles [64]. There are some reports that elaborated that IGF-1 is associated with an increase in the muscle mass and muscle fiber area, even inhibiting osteopenia [65,66]. The same finding can be observed in Figure 2 and Figure 6. It was speculated the IGF-1 function is partly affected by inflammation, oxidative stress, and diabetes [34,35]. In this study, treating the ageing db/db mice with Native Collagen II decreased oxidative stress (Figure 4), leading to an increased IGF-1 content (Figure 5). This finding was first reported in this study. This pathway might be the mechanism for an improvement of Native Collagen II in bone impairment.

The indirect effect of muscles on bone health was carried out through glucose metabolism. Skeletal muscles are recognized as the main site for insulin-mediated glucose disposal and energy metabolism. Muscle wasting can exacerbate insulin resistance, and higher muscle mass and strength are associated with a lower level of insulin resistance [67]. On the molecular level, skeletal homeostasis is linked to insulin sensitivity through nuclear receptor peroxisome proliferator-activated receptor (PPAR)γ [68]. IR increases the FOXO1 expression through PI3-K/MAPK, which could regulate osteoblast proliferation through ATF4 and p53 signaling [69]. Prolonged inflammation due to IR also stimulates the expression of proapoptotic genes such as the bcl-2-like protein (Bax). This reduces the expression of genes that stimulate osteoblast formation, such as the Fos-related antigen (FRA-1) and the Runt-related transcription factor (RUNX2), resulting in decreased bone formation. In T2DM, other proteins such as AGEs, proinflammatory cytokines, and ROS are increased [70]. Native Collagen II decreased oxidative stress both in the muscles and the serum (Figure 5 and Figure 6), indicating partly improved muscle IR (Figure 1), which was speculated to stimulate membrane translocation of GLUT4 through skeletal muscle phosphorylated PI3K protein expression, thereby increasing skeletal muscle glucose intake, leading to IR improvement [71]. Native Collagen II increased IGF-1 production by decreasing oxidative stress in the muscle to improve muscle foundation, glucose metabolism, and insulin signaling, thereby leading to bone formation.

On the other hand, hyperglycemia leads to the accelerated formation of AGEs and inflammation cytokines, inhibiting osteoblasts [72]. Therefore, inflammation and oxidative stress were speculated to be trigger factors of aggravating bone quality impairment. This study also found that Native Collagen II improved oxidative stress in the serum, which likely led to decreased AGE formation and cross-linking with collagen fibers. Interfering with the development and function of osteoblasts by upregulating the cell surface receptor for advanced glycation end products (RAGE) [14], these receptors increase the production of proinflammatory cytokines, which may feed a cycle of increased bone resorption and chronic inflammation [73].

**Figure 8:**
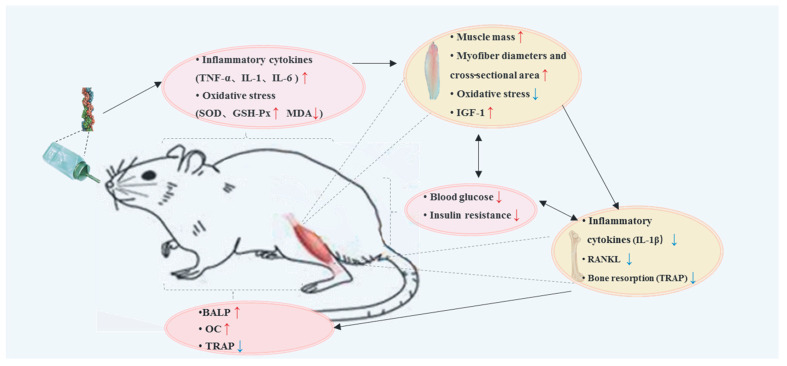
Graphical summation of the effect of Native Collagen II on impaired bone improvement and its likely mechanism.

Figure 4 shows that TNF-α, IL-6, and IL-1β levels in the serum were significantly decreased in the UC group. In previous cases of osteoarthritis, Native Collagen II was documented to promote a significant reduction in inflammation [74]. Figure 4, Figure 5 and Figure 7 show the relationship between inflammation and bone resorption (TRAP). Therefore, the osteoclasts overactivated by inflammation play a vital role in imbalanced bone metabolism [75]. Thus, inhibiting osteoclastogenesis through suppressing inflammation is an important strategy for improving bone degeneration [76]. Correspondingly, the positive expression of Il-1β in the CP group was decreased compared with that in the AM group (Figure 6A). IL-1β, IL-6, and TNF-α, common proinflammatory cytokines, can stimulate osteoclast activity with the macrophage colony-stimulating factor (M-CSF) and its receptor, c-Fms, which modulate the pool of available precursor cells for differentiation via the actions of RANKL [44]. These osteoclast-activating factors interact with a proven final common mediator of osteoclast differentiation and activation, receptor activator of nuclear factor-kB (RANK) and its functional ligand (RANKL). RANK is a membrane-bound TNF receptor expressed on osteoblast precursor cells that recognizes RANKL through direct cell–cell interactions, an essential process for the differentiation of osteoclasts from their precursor cells. RANKL, an essential factor for osteoclast differentiation and function, is also expressed by lymphocytes and synovial fibroblasts and may mediate bone loss associated with inflammatory conditions [37], which promotes the bone-resorbing activity of osteoclasts and prolongs their survival [38]. Similarly, the immunohistochemical analysis showed RANKL had a higher expression in the AM group, and low expression was observed in the CG and UC groups. The same tendency was shown in TRAP expression, which means that Native Collagen II decreased RANKL and TRAP expression. In this study, Native Collagen II decreased bone resorption by inhibiting serum cytokines, as well as IL-1 and IL-6 expression, and then inhibited the expression of RANKL and TRAP. In this study, it was speculated that Native Collagen II could improve bone impairment due to increasing bone mineralization and formation, decreasing bone resorption, which partly resulted from muscle function and IR improvement through decreasing oxidative stress and inflammation (Figure 8). This conclusion was partly proved in other studies [28,77].

This study has some limitations. First, the exact mechanisms of action of Native Collagen II on the bones impaired by T2DM will be explored in future in vitro and in vivo studies. Second, senescence-accelerated mice (SAM) will be adopted as the experimental model, especially to demonstrate ageing characteristics from a certain aspect, such as SAMP6 and SAMP10, and to explore the possible mechanisms of Native Collagen II in delaying and improving degenerative bone diseases.

## 5. Conclusions

In order to discover effective preventive measures for bone impairment in ageing coupled with T2DM, Native Collagen II was administered in the ageing db/db mice during a period of 3 months. The microarchitecture, mechanical properties, and bone metabolism were evaluated. In order to explore the likely mechanism of action of Native Collagen II, the T2DM disease status, biomarkers, and the gastrocnemius function were analyzed. The results showed that the Native Collagen II intervention elevated BMD and the biomechanical parameters. Our results also demonstrated that Native Collagen II increased bone mineralization and formation and decreased bone resorption. On the one hand, this function was partly due to decreasing inflammation, leading to inhibited RANKL and TRAP expression. On the other hand, Native Collagen II also improved oxidative stress in both the serum and the gastrocnemius muscle, leading to IGF-1 secretion, which facilitated bone formation and muscle growth, ultimately leading to improved IR. Of course, the improvement of hyperglycemia and IR due to Native Collagen II could decrease inflammation and the oxidative stress status, which feed an indirect cycle of bone impairment.

## Figures and Tables

**Table 1:** The bone biomechanical parameters of the femur in the five groups.

Groups	Maximum Load (N)	Energy to Ultimate Load (J)	Young’s Modulus (MPa)	Stiffness (N/mm)	Breaking Energy (J/m^2^)
AM	7.49 ± 1.49	0.0013 ± 0.001	775.18 ± 139.11	25.02 ± 9.19	692.99 ± 127.45
NC	9.69 ± 2.11	0.0037 ± 0.001 ^a^	1205.54 ± 371.03 ^a^	54.85 ± 13.53 ^a^	1605.45 ± 257.17 ^a^
YM	9.89 ± 1.88	0.0045 ± 0.001 ^a^	1190.34 ± 11.49 ^a^	43.32 ± 2.77 ^a^	1136.68 ± 22.99 ^a,b^
CG	15.44 ± 3.84 ^a,b^	0.0058 ± 0.002 ^a^	1603.71 ± 102.08 ^a,b,c^	53.11 ± 12.82 ^a^	1572.01 ± 312.61 ^a,c^
UC	18.01 ± 3.00 ^a,b,c^	0.0110 ± 0.003 ^a,b,c,d^	2875.55 ± 312.98 ^a,b,c,d^	79.94 ± 15.04 ^a,b,c,d^	2210.94 ± 15.09 ^a,b,c,d^

The data are expressed as the means ± SD, *n* = 5; ^a^ *p* < 0.05 versus the AM group, ^b^ *p* < 0.05 versus the NC group, ^c^ *p* < 0.05 versus the YM group, ^d^ *p* < 0.05 versus the CG group.

**Table 2:** Amino acid composition of Native Collagen II.

Amino Acid	Content (g/100 g)	Amino Acid	Content (g/100 g)
Asp	4.15	Val	2.05
Glu	8.72	Met	1.15
Ser	1.36	Phe	1.50
His	0.60	Ile	1.32
Gly	11.72	Leu	2.72
Thr	1.81	Lys	2.15
Arg	4.95	Pro	3.63
Ala	4.6	Hydroxyproline	8.26
Tyr	0.46		

## Data Availability

The data presented in this study are available on request from the corresponding author. The data are not publicly available due to graduation thesis based on this relevant research results is still in the confidentiality period.

## Supplementary Materials

The following are available online, Figure S1: The image of Native Collagen II with transmission electron microscope; Figure S2: The infrared spectroscopy of Native Collagen II; Figure S3: The food intake of db/db mice and db/m mice during the whole life in the experiments; Figure S4: The weight of db/db mice and db/m mice during the whole life in the experiments; Figure S5: The FBG of db/db mice and db/m mice during the whole life in the experiments.

## Sample Availability

No Samples of the compounds are available from the authors. All the compounds were bought.
