# Correction: Fan et al. Native Collagen II Relieves Bone Impairment through Improving Inflammation and Oxidative Stress in Ageing db/db Mice. *Molecules* **2021**, *26*, 4942

**DOI:** 10.3390/molecules27020571

**Published:** 2022-01-17

**Authors:** Rui Fan, Yuntao Hao, Xinran Liu, Jiawei Kang, Jiani Hu, Ruixue Mao, Rui Liu, Na Zhu, Meihong Xu, Yong Li

**Affiliations:** Department of Nutrition and Food Hygiene, School of Public Health, Peking University, Beijing 100191, China; fanruirf@bjmu.edu.cn (R.F.); haoyuntaolly@163.com (Y.H.); liuhappy07@163.com (X.L.); kangjwdt@163.com (J.K.); hujiani95@163.com (J.H.); rx334@163.com (R.M.); liuruipku@163.com (R.L.); summer920503@163.com (N.Z.); xumeihong@bjmu.edu.cn (M.X.)

The authors would like to correct spelling mistakes (undenatured type II collagen) in the title, as well as in the main manuscript including the tables and figures in the title paper [1]. This spelling error coincides with the US trademarked brand associated with a product manufactured by InterHealth. We provide, below, the corrected title as “Native Collagen II Relieves Bone Impairment through Improving Inflammation and Oxidative Stress in Ageing db/db Mice”, and the “Undenatured type II collagen” in the main manuscript including all the tables and figures, as well as in the supplementary materials, were also corrected as “native collagen II”. In addition, we deleted all the abbreviations “NC-II”, and used the full name as “native collagen II”. The change has no influence on the reported results. The original article has been updated.

The authors would like to apologize for any inconvenience caused to the readers by these changes.
