# Supplementary material for: Native Collagen II Relieves Bone Impairment through Improving Inflammation and Oxidative Stress in Ageing db/db Mice
Source: Molecules. 2021 Aug 15;26(16):4942. doi: 10.3390/molecules26164942 (PMC8400234; doi:10.3390/molecules26164942)
Supplement: Supplementary file 1 [file molecules-26-04942-s001.zip › molecules-1295939-Suppl-FC-correction needed.pdf]

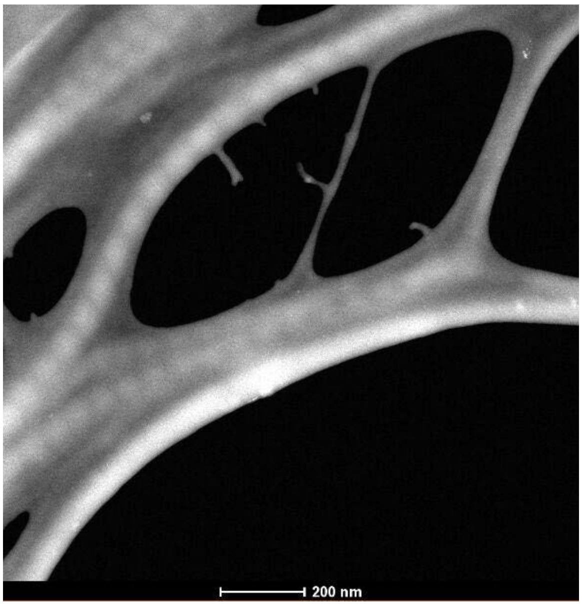

Figure S1. The image of native collagen II with transmission electron microscope

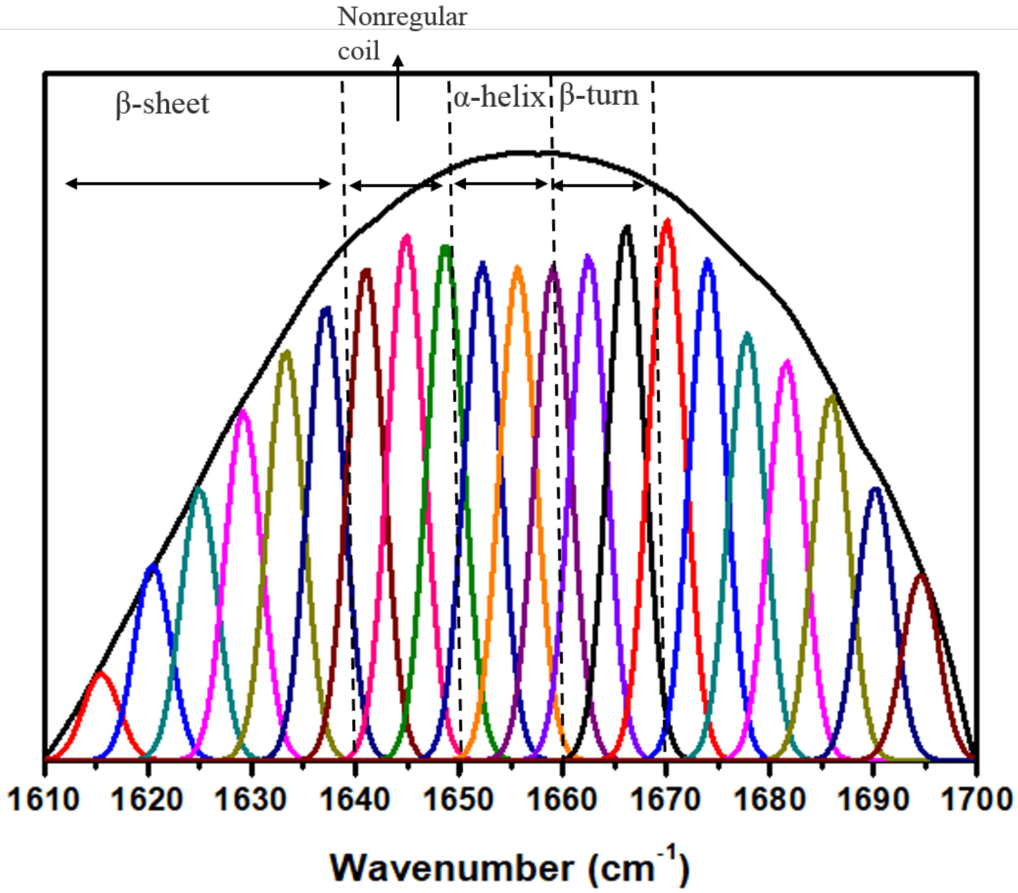

Figure S2. The infrared spectroscopy of native collagen II.

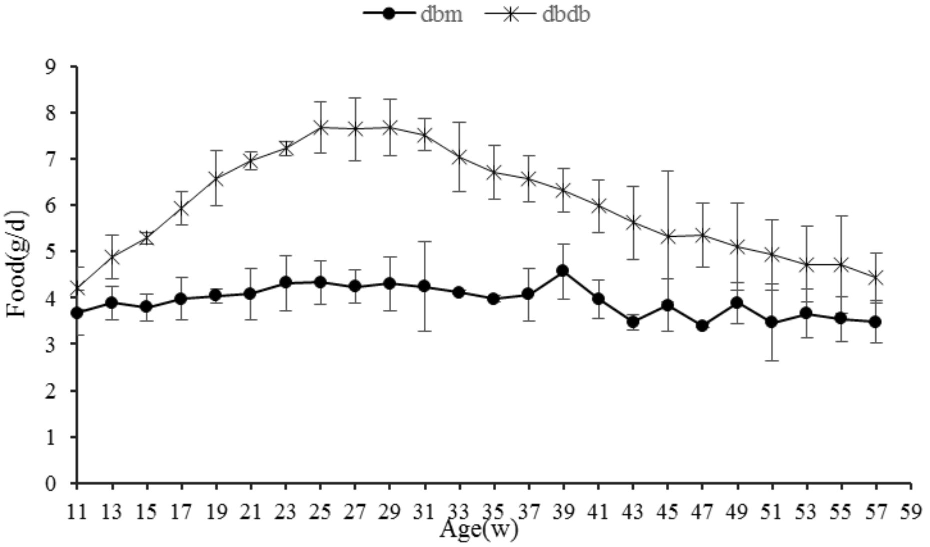

Figure S3. The food intake of db/db mice and db/m mice during the whole life in the experiments

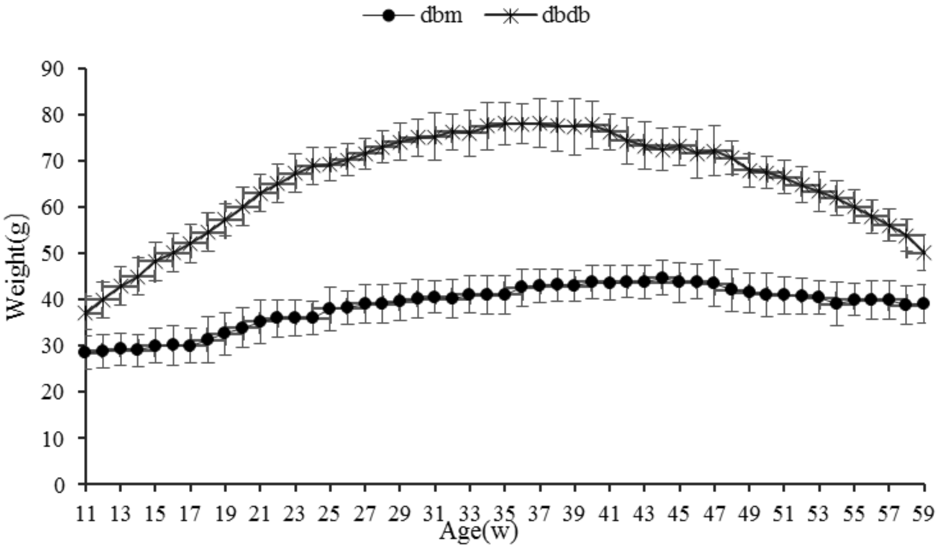

Figure S4. The weight of db/db mice and db/m mice during the whole life in the experiments

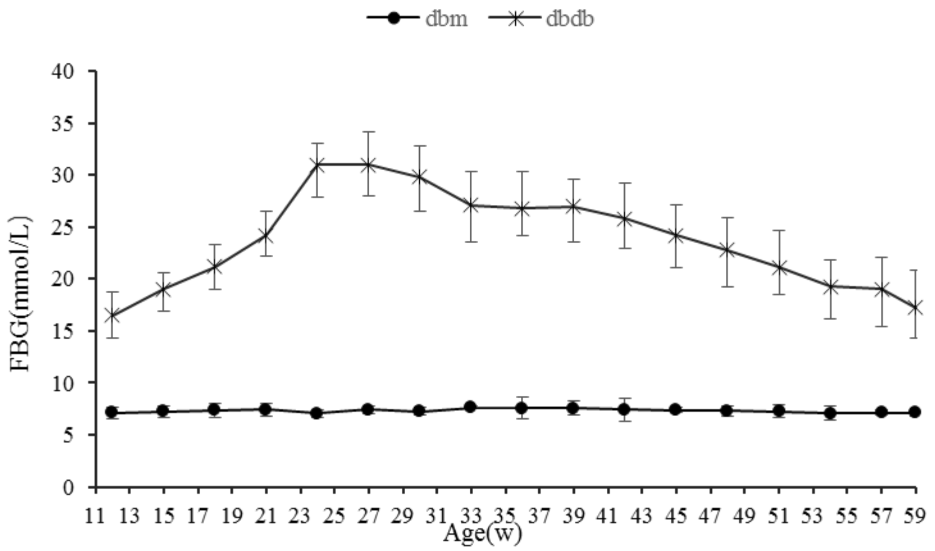

Figure S5. The FBG of db/db mice and db/m mice during the whole life in the experiments
